# Assessing the impact of socioeconomic distress on hospital readmissions after cardiac surgery

**DOI:** 10.1016/j.xjon.2024.07.002

**Published:** 2024-07-15

**Authors:** Mohamad El Moheb, Abhinav Kareddy, Steven Young, Matthew Weber, Sean Noona, Alexander Wisniewski, Anthony Norman, Zeyad Sahli, Raymond Strobel, Andrew Young, Jeffrey Rich, Abdulla Damluji, Mohammed Quader, Leora Yarboro, Nicholas Teman, Ourania Preventza

**Affiliations:** a Division of Cardiothoracic Surgery, Department of Surgery, University of Virginia, Charlottesville, Va; b Virginia Cardiac Services Quality Initiative, Virginia Beach, Va; c Inova Center of Outcomes Research, Inova Heart and Vascular Institute, Falls Church, Va; d Department of Cardiac Surgery, Virginia Commonwealth University, Richmond, Va

**Keywords:** CABG, readmission, quality of care, social determinants of health, socioeconomic status, community distress

## Abstract

**Background:**

The impact of socioeconomic distress on readmission rates following cardiac surgery has not been studied. We hypothesized that patients living in distressed communities would have a higher 30-day readmission rate after cardiac surgery compared to those living in less distressed communities.

**Methods:**

Patients undergoing isolated coronary artery bypass grafting (CABG) between 2016 and 2023 within a regional collaborative were identified. The Distressed Communities Index (DCI) and Area Deprivation Index (ADI) were used to measure socioeconomic distress. Two logistic regression models were performed to evaluate 30-day readmission rates: one incorporating ADI and the other including DCI. Models were adjusted for the Society of Thoracic Surgeons (STS) Predicted Risk of Mortality (PROM) score, postoperative complications, length of stay (LOS), year of surgery, and discharge disposition.

**Results:**

A total of 16,369 patients were included, of whom 10% were readmitted within 30 days of discharge. Readmitted patients were more likely to be female (32% vs 23.3%) and to develop postoperative complications (47% vs 35%) and less likely to be discharged to home (70.6% vs 83.5%; *P* < .001 for all). On multivariable analysis, STS PROM score, postoperative complications, prolonged LOS, and discharge to a facility or leaving against medical advice were predictive of higher readmission rates. Socioeconomic distress was not an independent predictor of readmission in the model that used DCI (odds ratio [OR], 0.93; 95% confidence interval [CI], 0.76-1.15) or in the model that used ADI (OR, 1.17; 95% CI, 0.83-1.64).

**Conclusions:**

In patients undergoing CABG, increasing socioeconomic distress does not predict higher 30-day readmission rate. Other factors, such as discharge location, have a greater impact on readmission rate.

## Graphical Abstract

**Figure:**
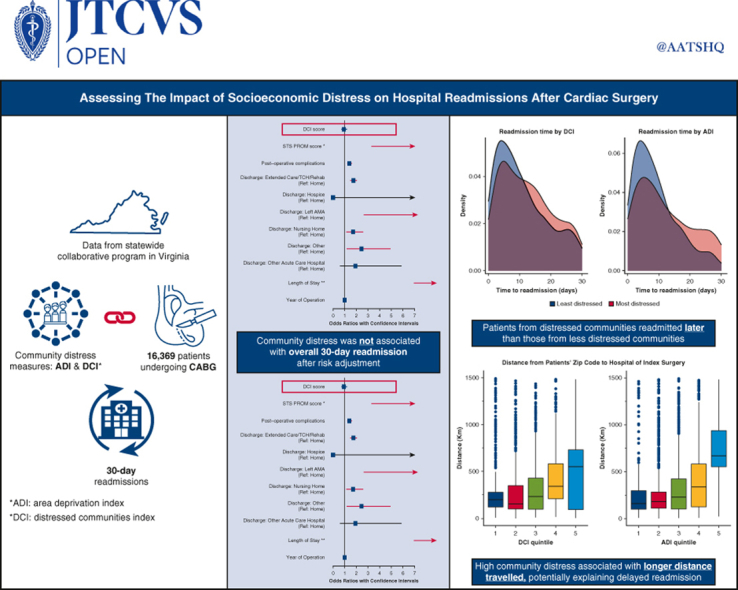

**Figure:**
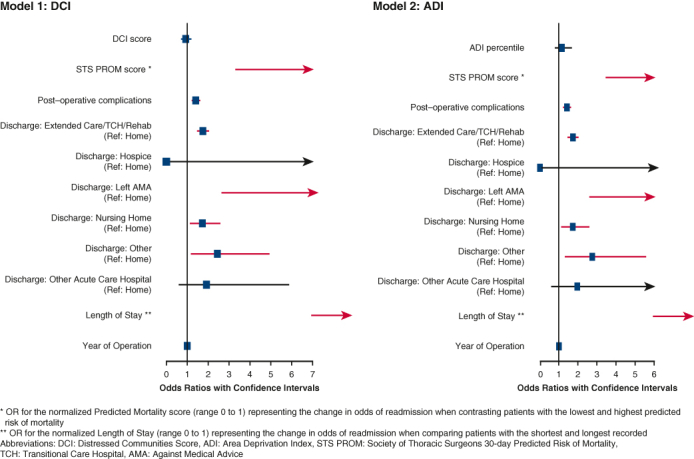
Influence of community distress levels on hospital readmission rates.

**Central Message**

After controlling for perioperative risk factors, patients from socioeconomically distressed communities undergoing coronary artery bypass grafting surgery are not at increased odds of 30-day readmission following hospital discharge.

**Perspective**

We investigated the impact of socioeconomic distress on risk-adjusted readmission after coronary artery bypass grafting surgery and found that socioeconomic distress does not predict overall 30-day readmissions. However, patients from less distressed communities had a higher early postoperative readmission rate compared to the more evenly distributed readmission timing observed in patients from increasingly distressed communities.

Coronary artery bypass grafting (CABG) is the most frequently performed cardiac surgery in the United States.^1^ Hospital readmission after CABG is of alarming proportions, with approximately 15% of patients readmitted within 30 days of discharge and 25% readmitted within 90 days.^2^ This constitutes an important economic burden and contributes to the continued rise in national healthcare expenditures.^3^, ^4^, ^5^ Beyond financial costs, readmissions disrupt patients' lives, limiting their capacity to work, care for family, or participate in community activities. Readmissions also lead to prolonged hospitalizations, increased stress, higher mortality risks, and diminished quality of life.^5^, ^6^, ^7^

In response to this problem, in 2012 the Centers for Medicare and Medicaid Services instituted the Hospital Readmissions Reduction Program, which imposes financial penalties on hospitals with excessive 30-day readmissions.^5^^,^^8^, ^9^, ^10^ Hospitals have subsequently adopted various clinically focused interventions to mitigate readmissions rates, including patient-needs assessments, multidisciplinary rounding teams, follow-up visits, and patient education.^9^^,^^10^ Despite these interventions, research into how social determinants of health, particularly socioeconomic distress, affect readmission rates is scarce. This gap in knowledge exists even as evidence increasingly shows that socioeconomic distress raises the risk of morbidity and mortality following cardiac surgery.^11^^,^^12^

The Area Deprivation Index (ADI) and Distressed Communities Index (DCI) have emerged as comprehensive measures of community distress, replacing traditional indicators of socioeconomic status (SES) such as education, income, and insurance.^12^, ^13^, ^14^ These indices have been shown to predict operative morbidity and mortality after cardiac surgery.^11^^,^^12^ With approximately 400,000 CABG procedures performed annually, understanding how socioeconomic distress affects readmission risk is essential.^1^ This study aimed to explore the association between socioeconomic distress, as measured by ADI and DCI, and risk-adjusted 30-day readmission rates after CABG. Given previous findings that individuals from distressed communities face worse postoperative outcomes following CABG, we hypothesized that patients from distressed communities are more likely to be readmitted within 30 days of discharge compared to those from less distressed communities.

## Methods

### Study Population

In this retrospective cohort study, we analyzed all patients who underwent isolated CABG between January 1, 2016, and January 1, 2023, who were included in the Virginia Cardiac Services Quality Initiative (VCSQI). The VCSQI is a prospectively maintained database (ARMUS Corporation) that captures Society of Thoracic Surgeons (STS) demographic, preoperative, clinical, and 30-day outcomes data on patients who undergo a cardiac surgical procedure at 17 participating institutions.^15^ Patients were excluded if they died during the initial hospitalization, died outside of the hospital within 30 days of discharge, or had missing readmission or ZIP code data. All identifying patient information was removed from the data, and the study was granted exemption by the University of Virginia Institutional Review Board (protocol 23305; deemed exempt on July 14, 2021).

### Socioeconomic Status

SES was first measured using the DCI. The DCI was developed by the Economic Innovation Group and is available for all ZIP codes with more than 500 residents, capturing 99% of the American population.^16^ It is a composite score based on 7 metrics obtained from the 5-year estimates from the American Communities Survey 2014 and the Census Bureau County and ZIP Code Business Patterns. These metrics are no high school degree, housing vacancy rate, adults not working, poverty rate, median income ratio, change in employment, and change in business establishments. The variables are evenly weighted and used to calculate a ZIP code's rank compared with its geographic peers, and then normalized to obtain a raw distress score that ranges from 0 (no distress) to 100 (severe distress). To mitigate potential limitations of relying on a single index, SES also was measured using the ADI. The ADI was developed by the University of Wisconsin School of Medicine and Public Health and uses 17 American Community Survey variables from the US Census comprising the domains of income, education, employment, and housing quality to stratify geographic areas based on socioeconomic disadvantage.^17^ The DCI and ADI indices were linked to the VCSQI data using patients' ZIP codes.

### Study Endpoints

The primary outcome of our study was readmission within 30 days of discharge from the index surgery hospitalization. The secondary endpoint was timing of readmission.

### Statistical Analysis

All continuous variables were reported as mean with standard deviation; categorical variables, as frequency with percentage. For univariate analyses, patients were stratified by national quintiles of DCI and ADI, allowing for an interpretable comparison between individuals at the high and low extremes of the distress scores. Continuous variables were compared using the independent-samples *t* test, and categorical variables were compared using the χ^2^ test. Pearson's correlation coefficient was calculated to measure the degree of agreement between DCI and ADI scores. Two multivariable logistic regression models, one incorporating ADI and the other with DCI, were performed to evaluate overall 30-day readmission rates. Two multivariable Cox proportional hazards models were built to evaluate the timing of readmissions. Models were adjusted for the STS Predicted Risk of Mortality (PROM) score, postoperative complications, hospital length of stay (LOS), year of surgery, and discharge disposition. The ADI and DCI were treated as continuous variables in the multivariable models to maintain data granularity and capture more nuanced relationships, and percentiles were converted to normalized scores ranging from 0 (least distress) to 1 (most distress). Random effects were controlled based on the hospital of surgery. The rate of missing data for the independent variables was <5%; therefore, we excluded these observations from the multivariable analysis and performed a complete case analysis. All tests were 2-sided, and a *P* value < .05 served as the threshold for significance. Data analyses were performed using R version 4.3.2 (R Foundation for Statistical Computing).

### Secondary Analyses

We conducted 4 additional analyses. First, a subgroup analysis was performed for patients discharged to home to determine whether distress score was predictive of 30-day readmission in this cohort. Second, despite the STS PROM score accounting for race, we performed a sensitivity analysis that explicitly included race as a variable in the multivariable model. This analysis focused specifically on comparing 30-day readmission rates between non-Hispanic black and white patients, with other races excluded, to assess potential differences when adjusting for additional variables. Third, a similar sensitivity analysis for sex was conducted in which sex was explicitly included in the multivariable model to investigate potential sex-based differences in readmission rates. Finally, a sensitivity analysis was performed examining the impact of distance to the nearest hospital on readmission rates and its interaction with distress score.

## Results

### Study Population

A total of 16,369 eligible patients were included, of whom 10% were readmitted within 30 days of discharge. The mean (SD) age of the cohort was 65.4 (9.9) years, and 76% were male (Table 1). The most common reasons for readmission were congestive heart failure (11%), arrythmia/heart block (7%), gastrointestinal problems (7%), pleural effusion necessitating intervention (6%), and pulmonary embolism (5%). Compared to patients who were not readmitted, those who were readmitted were more likely to be female (32% vs 23%; *P* < .001); had higher rates of comorbidities including diabetes (58% vs 49%; *P* < .001) chronic lung disease (37% vs 28%; *P* < .001), and heart failure (34% vs 25%; *P* < .001); were more likely to develop postoperative complications (47% vs 35%: *P* < .001); and were less likely to be discharged to home (71% vs 84%; *P* < .001).

**Table 1:** Characteristics of study population by readmission status

Characteristic	Not readmitted (N = 14,746)	Readmitted (N = 1633)	*P* value
Preoperative			
Age, y, mean (SD)	65.3 (9.9)	66.3 (10.0)	<.001
Male sex, n (%)	11,304 (76.7)	1111 (68.0)	<.001
Race, n (%)			.01
White	11,944 (82.2)	1277 (79.3)	
Black	1924 (13.2)	247 (15.3)	
Asian	378 (2.6)	41 (2.5)	
Other	287 (1.9)	45 (2.8)	
Ethnicity, non-Hispanic, n (%)	13,664 (97.9)	1506 (97.2)	.096
BMI, mean (SD)	30.1 (6.5)	31.2 (12.8)	<.001
Diabetes, n (%)	7174 (48.7)	951 (58.3)	<.001
Dialysis, n (%)	393 (2.7)	94 (5.8)	<.001
Hypertension, n (%)	13,260 (89.9)	1508 (92.4)	.002
Current smoker, n (%)	3388 (23.0)	359 (22.0)	.371
Alcohol intake (drinks/wk), n (%)			<.001
Low (1-2)	11,166 (76.8)	1315 (82.0)	
Moderate (2-7)	1292 (8.9)	115 (7.2)	
Heavy (>8)	2082 (14.3)	174 (10.8)	
Cirrhosis, n (%)	68 (1.0)	6 (0.8)	.797
Immunosuppression, n (%)	518 (3.5)	78 (4.8)	.012
Cancer, n (%)	716 (4.9)	98 (6.0)	.05
Previous stroke, n (%)	1236 (8.4)	182 (11.1)	<.001
Sleep apnea, n (%)	3080 (21.2)	378 (23.6)	.028
Mediastinal radiation, n (%)	117 (0.8)	19 (1.2)	.156
Peripheral vascular disease, n (%)	2002 (13.6)	300 (18.4)	<.001
Chronic lung disease, n (%)	4139 (28.1)	606 (37.1)	<.001
Previous MI, n (%)	8289 (56.5)	1004 (61.9)	<.001
Heart failure, n (%)	3682 (25.0)	547 (33.5)	<.001
Arrythmia, n (%)	2254 (15.3)	347 (21.3)	<.001
STS predicted mortality score, mean (SD)	0.02 (0.03)	0.02 (0.03)	<.001
Operative			
Emergency surgery, n (%)	9167 (62.2)	1110 (68.0)	<.001
Perfusion time, min, mean (SD)	93.7 (38.5)	94.5 (38.7)	.424
Cross-clamp time, min, mean (SD)	69.5 (31.5)	68.5 (31.5)	.204
Postoperative			
Postoperative complications, n (%)	5166 (35.0)	768 (47.0)	<.001
Reoperation, n (%)	497 (3.4)	90 (5.5)	<.001
ICU readmission, n (%)	425 (2.9)	82 (5.0)	<.001
Discharge destination, n (%)			<.001
Home	12,306 (83.5)	1153 (70.6)	
Extended care/TCU/rehab	2180 (14.8)	420 (25.7)	
Hospice	4 (0.0)	0 (0.0)	
Left AMA	9 (0.1)	7 (0.4)	
Nursing home	167 (1.1)	39 (2.4)	
Other	47 (0.3)	10 (0.6)	
Other acute care hospital	18 (0.1)	4 (0.2)	
ICU LOS, h, mean (SD)	74.8 (86.1)	92.0 (109.4)	<.001
Hospital LOS, d, mean (SD)	9.9 (7.6)	12.3 (11.7)	<.001

*SD*, Standard deviation; *BMI*, body mass index; *MI*, myocardial infarction; *STS*, Society of Thoracic Surgeons; *ICU*, intensive care unit; *TCU*, transitional care unit; *AMA*, against medical advice; *LOS*, length of stay.

### Socioeconomic Distress

**Figure 1:**
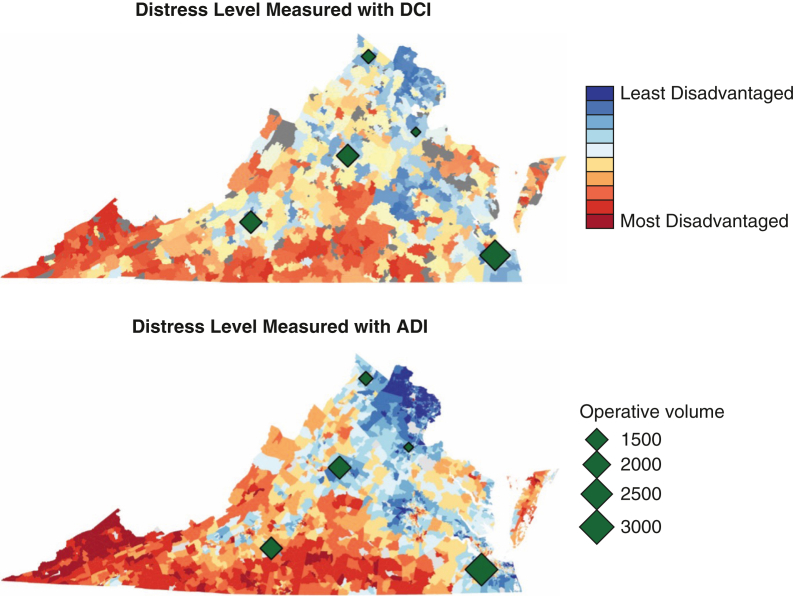
Distressed communities in Virginia as measured by the Distressed Communities Index (*DCI*) and Area Deprivation Index (*ADI*), superimposed with the location of the top 5 hospitals with the highest operative volumes.

We constructed maps depicting the distress levels of communities within Virginia, as measured by the DCI and ADI (Figure 1). The distributions displayed by the 2 indices were similar, indicating a high degree of concordance. The most distressed communities were concentrated in southwestern Virginia, whereas the more prosperous were in the northern region and along the coastline. Pearson's coefficient was 0.80, confirming a strong correlation between the 2 measures. The hospitals with the highest operative volume were generally located in areas of low DCI (Figure 1).

Table 2 summarizes the characteristics of patients living in the most distressed communities versus those in the least distressed communities. Patients living in the most distressed communities as measured by the DCI were more likely to be female (28% vs 22%; *P* < .001) and black (23% vs 8%; *P* < .001); had more comorbidities, including diabetes (54% vs 47%; *P* < .001), smoking (28% vs 18%; *P* < .001), and history of myocardial infarction (60% vs 52%; *P* < .001); had longer hospital stays (mean LOS, 10.7 [7.8] days vs 9.6 [9.3] days; *P* < .001); and were less likely to be discharged home (79% vs 86%; *P* < .001). There was no difference in postoperative complications between the 2 groups.

**Table 2:** Characteristics of patients in the highest and lowest quintiles of distress as assessed by the DCI and ADI

Characteristic	DCI			ADI		
	Least distressed (N = 3587)	Most distressed (N = 2372)	*P* value	Least distressed (N = 774)	Most distressed (N = 1272)	*P* value
Preoperative						
Age, y, mean (SD)	65.9 (9.9)	64.5 (9.8)	<.001	65.8 (9.8)	64.9 (9.6)	.054
Male sex, n (%)	2813 (78.4)	1711 (72.2)	<.001	636 (82.2)	940 (73.9)	<.001
Race, n (%)			<.001			<.001
White	2958 (84.2)	1749 (74.5)		539 (73.6)	1058 (84.0)	
Black	276 (7.9)	549 (23.4)		35 (4.8)	187 (14.9)	
Asian	182 (5.2)	18 (0.8)		124 (16.9)	0 (0.0)	
Other	99 (2.7)	32 (1.4)		34 (4.6)	14 (1.1)	
Ethnicity, non-Hispanic, n (%)	3278 (97.6)	2274 (98.4)	.047	713 (95.2)	1240 (99.1)	<.001
BMI, mean (SD)	29.92 (6.95)	30.49 (6.35)	.001	28.73 (5.58)	30.09 (5.63)	<.001
Diabetes, n (%)	1693 (47.3)	1285 (54.2)	<.001	344 (44.6)	676 (53.1)	<.001
Dialysis, n (%)	85 (2.4)	89 (3.8)	.003	19 (2.5)	45 (3.5)	.219
Hypertension, n (%)	3155 (88.0)	2182 (92.0)	<.001	647 (83.6)	1159 (91.1)	<.001
Current smoker, n (%)	654 (18.3)	664 (28.1)	<.001	125 (16.2)	344 (27.1)	<.001
Alcohol intake (drinks/wk), n (%)			<.001			<.001
Low (1-2)	2639 (74.7)	1913 (81.9)		532 (71.2)	1044 (83.1)	
Moderate (2-7)	577 (16.3)	225 (9.6)		134 (17.9)	114 (9.1)	
Heavy (>8)	319 (9.0)	199 (8.5)		81 (10.8)	98 (7.8)	
Cirrhosis, n (%)	16 (0.8)	7 (0.6)	.727	2 (0.8)	2 (0.4)	.903
Immunosuppression, n (%)	128 (3.6)	72 (3.0)	.295	22 (2.8)	42 (3.3)	.654
Cancer, n (%)	176 (4.9)	99 (4.2)	.211	48 (6.2)	50 (4.0)	.027
Previous stroke, n (%)	268 (7.5)	234 (9.9)	.001	58 (7.5)	112 (8.8)	.337
Sleep apnea, n (%)	733 (20.6)	464 (19.8)	.437	134 (17.3)	244 (19.3)	.286
Mediastinal radiation, n (%)	26 (0.7)	14 (0.6)	.644	7 (0.9)	13 (1.0)	.978
Peripheral vascular disease, n (%)	477 (13.3)	359 (15.2)	.047	101 (13.1)	162 (12.8)	.903
Chronic lung disease, n (%)	964 (26.9)	707 (29.8)	.015	270 (34.9)	348 (27.4)	<.001
Previous MI, n (%)	1869 (52.3)	1421 (60.4)	<.001	375 (48.5)	752 (59.5)	<.001
Heart failure, n (%)	922 (25.7)	593 (25.0)	.562	171 (22.1)	262 (20.6)	.455
Arrythmia, n (%)	621 (17.3)	358 (15.1)	.026	139 (18.0)	198 (15.6)	.176
STS PROM, mean (SD)	0.02 (0.03)	0.02 (0.03)	.158	0.02 (0.04)	0.02 (0.02)	.047
Operative						
Emergency surgery, n (%)	2205 (61.5)	1508 (63.6)	.107	473 (61.1)	762 (59.9)	.621
Perfusion time, min, mean (SD)	91.2 (38.0)	92.4 (36.5)	.215	90.38 (36.6)	94.06 (39.8)	.036
Cross-clamp time, min, mean (SD)	68.1 (32.3)	67.4 (29.9)	.391	65.9 (30.7)	67.59 (29.7)	.231
Postoperative						
Postoperative complications, n (%)	1250 (34.9)	876 (36.9)	.108	221 (28.6)	463 (36.4)	<.001
Reoperation, n (%)	111 (3.1)	87 (3.7)	.256	27 (3.5)	46 (3.6)	.977
ICU readmission, n (%)	98 (2.7)	76 (3.2)	.326	23 (3.0)	42 (3.3)	.777
Discharge destination, n (%)			<.001			<.001
Home	3073 (85.7)	1861 (78.5)		669 (86.4)	987 (77.7)	
Extended care/TCU/rehab	469 (13.1)	446 (18.8)		92 (11.9)	253 (19.9)	
Hospice	0 (0.0)	0 (0.0)		0 (0.0)	0 (0.0)	
Left AMA	3 (0.1)	3 (0.1)		0 (0.0)	3 (0.2)	
Nursing home	33 (0.9)	33 (1.4)		10 (1.3)	12 (0.9)	
Other	2 (0.1)	24 (1.0)		2 (0.3)	13 (1.0)	
Other acute care hospital	4 (0.1)	4 (0.2)		1 (0.1)	3 (0.2)	
ICU LOS, h, mean (SD)	74.7 (87.6)	81.8 (74.2)	.001	58.5 (100.7)	81.9 (75.3)	<.001
Hospital LOS, d, mean (SD)	9.6 (9.3)	10.7 (7.8)	<.001	9.40 (9.8)	10.3 (6.6)	.017

*DCI*, Distressed Communities Index; *ADI*, Area Deprivation Index; *SD*, standard deviation; *BMI*, body mass index; *MI*, myocardial infarction; *STS*, Society of Thoracic Surgeons; *PROM*, Predicted Risk of Mortality; *ICU*, intensive care unit; *TCU*, transitional care unit; *AMA*, against medical advice; *LOS*, length of stay.

When socioeconomic distress was measured using the ADI, patients living in the most distressed communities were more likely to be female and black and had more comorbidities, longer hospital LOS, and a lower likelihood of being discharged to home; however, unlike with the DCI, they had a higher rate of postoperative complications (36% vs 29%; *P* < .001). There was no difference in the reasons for 30-day readmissions between patients living in the most distressed communities and those living in the least distressed communities as measured by the DCI (*P* = .29) (Table E1).

**Figure E1:**
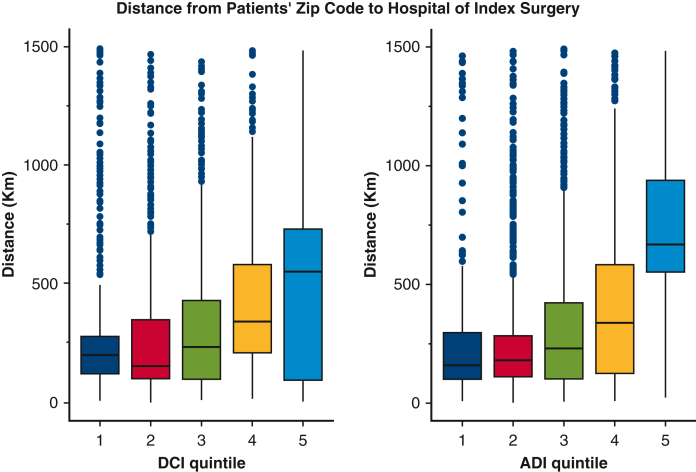
Boxplots of distance to hospital of index surgery from patients' ZIP codes, by Distressed Communities Index (*DCI*) and Area Deprivation Index (*ADI*) quintiles. The box's *lower* and *upper borders* represent the quartiles, and the *middle line* indicates the median. The whiskers indicate the range of nonoutliers, and the *dots* represent outliers.

**Figure E2:**
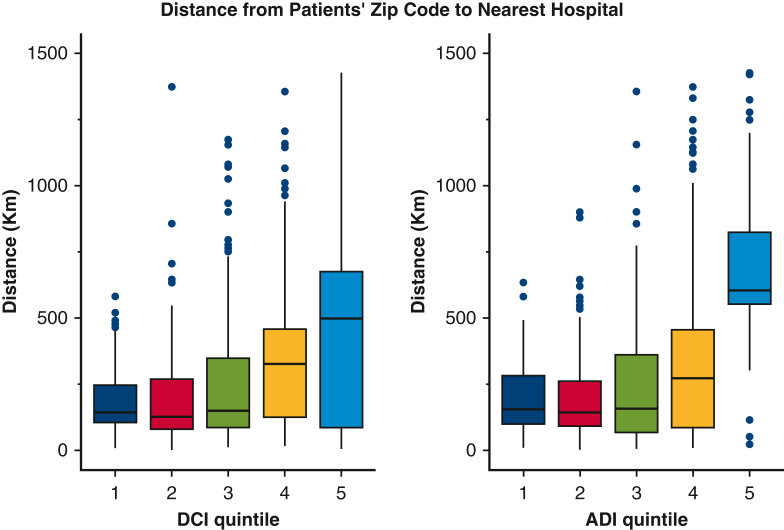
Boxplots of distance to the nearest Virginia Cardiac Services Quality Initiative (*VCSQI*) hospital from patients' ZIP codes, by Distressed Communities Index (*DCI*) and Area Deprivation Index (*ADI*) quintiles. The box's *lower* and *upper borders* represent the quartiles, and the *middle line* indicates the median. The whiskers indicate the range of nonoutliers, and the *dots* represent outliers.

Given the lack of specific data on readmission sites, access to care distances were estimated by calculating the distance between the patient's residential ZIP code and the locations of the hospital where the index surgery was performed and the nearest VCSQI hospital (Figures E1 and E2). Patients residing in distressed communities were found to live farther from both their surgery hospital and the nearest VCSQI hospital compared to patients from less distressed communities.

### Evaluation of Primary and Secondary Outcomes

**Figure 2:**
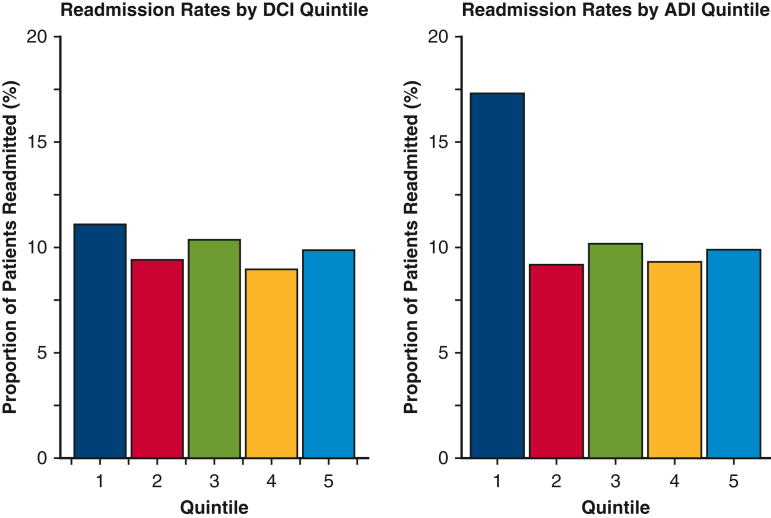
Unadjusted comparison of hospital readmission rates across Distressed Communities Index (*DCI*) and Area Deprivation Index (*ADI*) quintiles.

Unadjusted bivariate logistic regression analysis showed that DCI was significantly associated with increased risk of 30-day readmissions (*P* = .035), with a higher rate of readmission in patients living in the most prosperous DCI ZIP codes compared to those living the most distressed communities (11.1% for the first quintile, 9.4% for the second quintile, 10.4% for the third quintile, 9.0% for the fourth quintile, and 9.9% for the fifth quintile). Univariate analysis revealed a similar though more pronounced trend for ADI, with a significantly higher rate of 30-day readmission in patients living in the most prosperous ADI communities compared to all other groups (17.3% for the quintile, 9.2% for the second quintile, 10.2% for the third quintile, 9.3% for the fourth quintile, and 9.9% or the fifth quintile; *P* < .001) (Figure 2).

**Figure 3:**
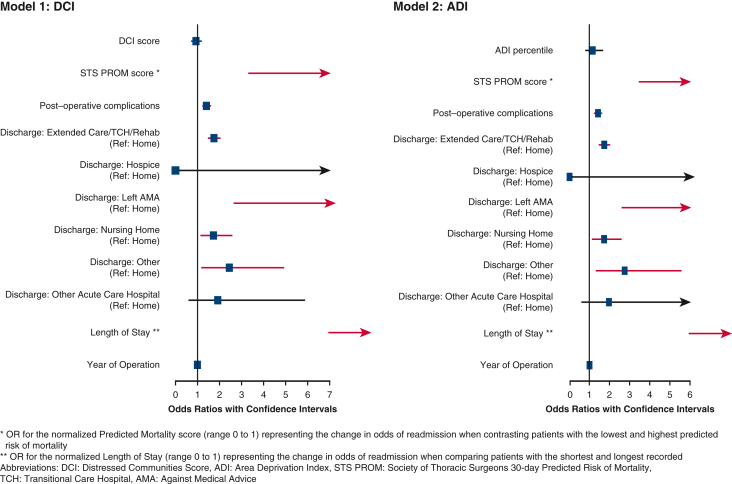
Forest plots of multivariable logistic regression models evaluating the influence of community distress levels, measured by the Distressed Communities Index (*DCI*) and the Area Deprivation Index (*ADI*), on hospital readmission rates.

On multivariable analysis, STS PROM score, postoperative complications, prolonged LOS, and discharge to an extended care facility, nursing home, or leaving against medical advice were predictive of higher 30-day readmission rate (Figure 3). However, socioeconomic distress was not an independent predictor of 30-day readmission in the model that used DCI (odds ratio [OR], 0.93; 95% confidence interval [CI], 0.76-1.15; *P* = .50) or in the model that used ADI (OR, 1.17; 95% CI, 0.83-1.64; *P* = .38). We examined the interaction between community distress scores and discharge location and found no significant interaction effect (*P* = .15-.98).

**Figure 4:**
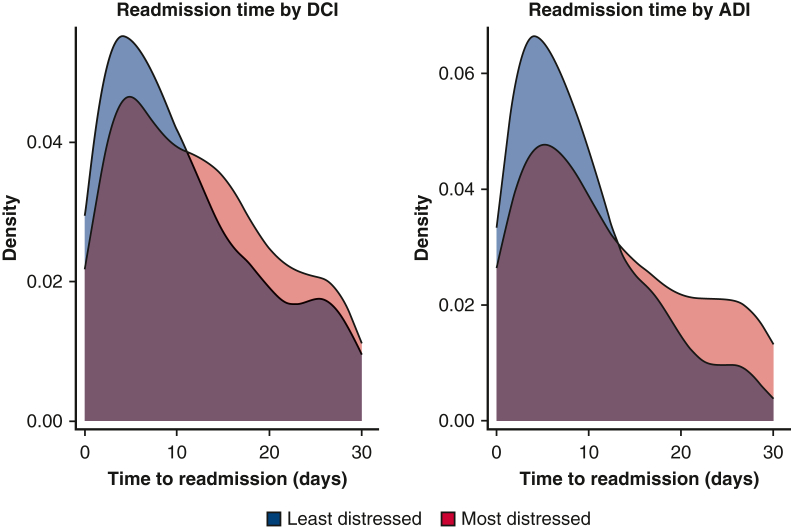
Density plot of time to readmission for patients in the most (*bottom* quintile) and least (*top* quintile) distressed communities as measured by Distressed Communities Index (*DCI*) and Area Deprivation Index (*ADI*).

**Figure 5:**
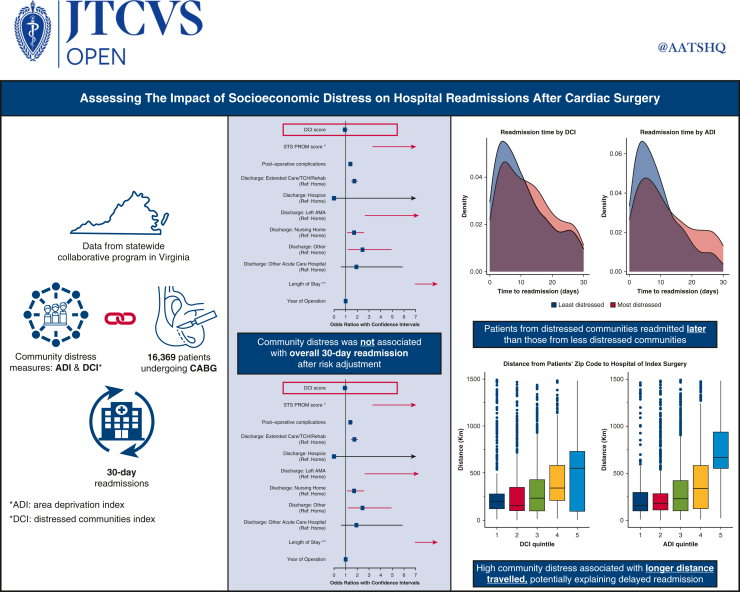
Graphical abstract.

Cox proportional hazard regression was performed to evaluate the relationship between timing to readmission and community distress level. When community distress was measured with the DCI, there was no association between distress score and time to readmission; however, when community distress was measured with the ADI, there was a statistically significant relationship between ADI score and timing to readmission (hazard ratio [HR], 0.73; 95% CI, 0.55-0.95), representing the change in hazard when comparing patients with the lowest versus highest ADI scores. Figure 4 illustrates the distribution of time to readmission between individuals in the highest versus lowest quintiles of the DCI and ADI. Patients from the least distressed communities tend to be readmitted sooner, with an initial surge in readmissions occurring shortly after discharge. In contrast, patients from more distressed areas exhibited a broader spread in their readmission times. A similar model was run in which insurance status was explicitly included in the multivariable Cox model, and the relationship between community distress and readmission rates remained significant (HR, 0.66; 95% CI, 0.35-0.97), whereas insurance status was not a significant predictor. Figure 5 provides a graphical abstract of the study.

### Subgroup and Sensitivity Analyses

In the subgroup analysis of patients discharged to home, neither the ADI nor the DCI was a significant predictor of increased 30-day readmission rate (*P* = .31 and .59, respectively). In the sensitivity analysis exploring racial disparities in outcomes between non-Hispanic black and white patients revealed no significant difference in 30-day readmission rate after risk adjustment (DCI model, *P* = .23; ADI model, *P* = .38). This persisted even when analyzing the subgroup of black and white patients living in the highest quintile of community distress (DCI model, *P* = .30; ADI model, *P* = .13). However, comparing outcomes by sex revealed a significantly higher odds of readmission for female patients compared to male patients (DCI model: OR, 1.46; 95% CI, 1.30-1.63; *P* < .001; ADI model: OR, 1.45; 95% CI, 1.29-1.63; *P* < .001). Finally, in our sensitivity analysis examining the impact of distance to the nearest hospital on readmission rates and its interaction with distress score, we found that the individual effects of community distress and distance were not statistically significant (OR, 1.01; 95% CI, 0.73-1.39 and OR, 3.69; 95% CI, 0.62-21.7, respectively), but their interaction was significant (OR, 0.075; 95% CI, 0.006-0.92), suggesting that patients from highly distressed communities who live farther from the hospital are less likely to be readmitted.

## Discussion

Hospital readmission rate serves as a critical indicator of surgical quality of care and is an important driver of increased healthcare costs. Using data from a statewide, multi-institutional collaborative registry, we aimed to determine whether the level of community distress predicts 30-day readmission following cardiac surgery. Our analysis focused exclusively on patients undergoing isolated CABG to minimize heterogeneity in the study population. Our findings show that community distress level, when measured with the DCI, does not independently predict increased 30-day readmission after risk adjustment. We further analyzed community distress using the ADI and also found no independent predictive value for higher 30-day readmission. However, our study identified other significant predictors of 30-day readmission, such as the STS PROM score, postoperative complications, hospital LOS, and, notably, the discharge location. Patients discharged to an extended care facility, rehab, or transitional care unit were 75% more likely to be readmitted within 30 days compared to those discharged to home. When considering only the subgroup of patients discharged to home, community distress levels still did not emerge as a predictor of 30-day readmission. Interestingly, we observed a sex-based disparity, with female patients more likely to be readmitted than their male counterparts, even after adjusting for other covariates. This finding is consistent with previous studies reporting worse postoperative outcomes for females following cardiac surgery.^18^^,^^19^

We subsequently analyzed the timing of readmissions and found that although the overall readmission rates across the 30-day period did not vary according to the logistic regression models, the timing of these readmissions posthospitalization did vary, as indicated by the HRs from the Cox regression model. Patients from low-distress communities showed a peak in readmission rates shortly after discharge, followed by a rapid decline. In contrast, readmission rates for patients from higher-distress communities were more evenly distributed over time, with a greater occurrence of readmissions later after discharge. This pattern indicates that readmissions tend to occur earlier in less distressed communities, whereas in more distressed communities, readmissions are spread out more consistently throughout the 30-day period.

These findings warrant cautious interpretation and should not be construed to suggest that patients from low-distress communities and high-distress communities experience similar outcomes. Charles and colleagues^12^ demonstrated that higher socioeconomic distress predicts increased mortality after CABG. Similarly, Mehaffey and colleagues^20^ reported a direct correlation between high DCI score and increased risk of morbidity and mortality following CABG.^20^ Therefore, the absence of observable differences in 30-day readmission rates may be related to the higher likelihood of patients from distressed communities failing to reach the hospital in time, thus succumbing more frequently to their complications compared to those from more affluent neighborhoods. Although it is difficult to confirm this hypothesis in a retrospective study, our findings hint at this possibility, given the higher readmission rate in the early postoperative period among patients from prosperous neighborhoods. This is supported by data showing that individuals residing in distressed communities have longer travel distances to both the index surgery hospital and the nearest VCSQI hospital. Such extended travel distances could pose a significant barrier to accessing timely care for patients from lower socioeconomic backgrounds.

Notably, the hospitals with the highest surgical volumes in our study were largely located in areas of lower distress, which may contribute to prompt care access for patients of higher SES experiencing postoperative complications, potentially leading to better outcomes. In contrast, patients of lower SES contending with longer distances might experience prehospital mortality or delayed admissions when presenting with advanced conditions.

Similarly, the absence of a detectable difference in readmission rates between black and white patients in the sensitivity analysis should not be interpreted as evidence of comparable postoperative outcomes following CABG. Research into long-term outcomes after cardiac surgery indicates that black patients typically experience worse outcomes than white patients.^21^ Our analysis was confined to 30-day readmissions, however. When assessing the distribution of readmission timing, especially during the later postdischarge period, we observed a higher proportion of patients from distressed communities. This suggests that an analysis extending to 60-day or 90-day readmissions potentially could reveal disparate outcomes in the 2 groups.

The results from the ADI and DCI analyses were in agreement, except for the survival analysis, in which only the ADI was predictive of the timing of readmission. Although these measures are similar, they evaluate distinct facets of community distress, as evidenced by a correlation coefficient of 0.8, which indicates strong, but not perfect, correlation. Although the ADI uses more detailed census tract–level data, providing higher sensitivity to detect variations in distress levels, the DCI covers a wider range of economic indicators, providing a broader perspective on community economic health and distress.

Previous studies also have identified discrepancies between ADI and DCI outcomes. For instance, Ghirimoldi and colleagues^13^ found that only the ADI, and not the DCI, was associated with 30-day readmission rate following colorectal surgery; however, they showed that higher ADI scores were linked to increased readmission rates, contrary to our results. Research in orthopedic surgery similarly reported higher readmission rates among patients from areas with high social vulnerability.^22^ This difference in findings likely is related to incomplete risk adjustment in prior studies, as well as the high mortality rate in cardiac surgery, which disproportionately affect individuals from distressed communities.^20^ Consequently, these individuals often are not included in analyses. Our multivariable logistic regression model accounted for baseline surgical risk using the STS PROM score and factors related to patients' postoperative course, including hospital LOS and complications. In contrast, Ghirimoldi and colleagues^13^ adjusted only for baseline frailty and postoperative complications, omitting other potential confounders such as comorbidities and LOS, which we found to be associated with higher distress levels and are known predictors of rehospitalization.^11^^,^^23^ Similarly, studies in other surgical fields also have failed to account for the patient's postoperative course.^14^^,^^24^^,^^25^

Interestingly, Johnson and colleagues demonstrated that the ADI was predictive of rehospitalization in cardiac patients.^26^ A notable difference from our study is their inclusion of all patients admitted for heart failure, myocardial infarction, or atrial fibrillation via the emergency department, which implies a focus on medically managed admissions. Despite this, the observed disparities in posthospitalization outcomes between medically and surgically managed cardiac patients merit further examination. One possible reason for these differences might be the stringent selection criteria for surgical intervention. Typically, surgical candidates are more proactive about their health care, enjoy better access to referral systems, and are more likely to comply with follow-up care. Thus, patients undergoing surgery constitute a distinct subset of the broader cardiac patient population, regardless of SES.

Our study is not without limitations. First, the retrospective design may introduce unmeasured confounding variables. Second, the ADI and DCI serve as indirect indicators of an individual's SES, providing community-level rather than patient-specific data, and as such, there might be a discrepancy between a patient's SES and that of their community. Third, our study could be subject to selection bias, potentially only including patients who accessed medical centers through referral. Given that SES is a significant obstacle to accessing quality healthcare, the most distressed patients needing cardiac surgery might not have been referred to or reached the hospital for surgery. Fourth, there is a possibility that patients might have relocated during the study period, which could have led to incomplete capture of readmission data. Furthermore, readmissions to hospitals within different systems, such as VA hospitals, might not be included. Finally, given that all participating centers are located within the state of Virginia, our findings might not be generalizable to the broader population.

## Conclusions

In patients undergoing CABG, socioeconomic distress does not independently predict higher risk of hospital readmission within 30 days of discharge. Other factors, such as the patient's preoperative risk, postoperative recovery course, and discharge destination, have a more significant influence on readmission. Although overall readmission rates did not vary, patients from less socioeconomically distressed communities had a higher rate of early postoperative readmission, indicating potential delays in care for lower SES groups. Further research should focus on targeted interventions that address both the medical and social factors affecting readmission, focusing in particular on enhancing early postdischarge support and access to care for patients of lower SES.

### Webcast

You can watch a Webcast of this AATS meeting presentation by going to: https://www.aats.org/resources/evaluating-the-impact-of-socio-7575.

**Figure:**
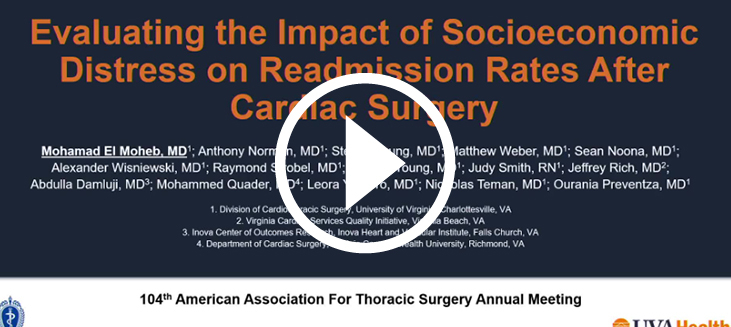

## Conflict of Interest Statement

The authors reported no conflicts of interest.

The *Journal* policy requires editors and reviewers to disclose conflicts of interest and to decline handling or reviewing manuscripts for which they may have a conflict of interest. The editors and reviewers of this article have no conflicts of interest.

## Figures and Tables

**Table E1:** Reasons for readmission of highest distressed versus lowest distressed communities as measured by the DCI

Reason for readmission	1st DCI quintile (N = 398), n (%)	5th DCI quintile (N = 234), n (%)	*P* value
Angina	8 (2.0)	5 (2.1)	.289
Anticoagulation complication – pharmacologic	2 (0.5)	2 (0.9)	
Arrhythmia/heart block	36 (9.2)	16 (6.9)	
Blood pressure (hypertension or hypotension)	3 (0.8)	4 (1.7)	
Chest pain, noncardiac	7 (1.8)	7 (3.0)	
Congestive heart failure	41 (10.4)	31 (13.3)	
Coronary artery/graft dysfunction	1 (0.3)	3 (1.3)	
Depression/psychiatric issue	1 (0.3)	0 (0.0)	
Deep vein thrombosis	4 (1.0)	2 (0.9)	
Electrolyte imbalance	5 (1.3)	2 (0.9)	
Failure to thrive	0 (0.0)	1 (0.4)	
Gastrointestinal issue	28 (7.1)	11 (4.7)	
Infection, conduit harvest site	3 (0.8)	5 (2.1)	
Infection, deep sternum/mediastinitis	18 (4.6)	11 (4.7)	
Mental status changes	2 (0.5)	1 (0.4)	
Myocardial infarction	9 (2.3)	6 (2.6)	
Myocardial infarction and/or recurrent angina	3 (0.8)	0 (0.0)	
Other, nonrelated readmission	31 (7.9)	15 (6.4)	
Other, related readmission	51 (13.0)	34 (14.6)	
Pleural effusion	23 (5.9)	5 (2.1)	
Pericardial effusion and/or tamponade	9 (2.3)	4 (1.7)	
Pleural effusion requiring intervention	36 (9.2)	17 (7.3)	
Pneumonia	13 (3.3)	11 (4.7)	
Renal insufficiency	2 (0.5)	0 (0.0)	
Respiratory complications, other	13 (3.3)	8 (3.4)	
Sepsis	10 (2.5)	3 (1.3)	
Stroke	9 (2.3)	3 (1.3)	
Transient ischemic attack	1 (0.3)	0 (0.0)	
Transfusion	3 (0.8)	0 (0.0)	
Vascular complication, acute	1 (0.3)	0 (0.0)	
Wound, other (drainage, cellulitis)	16 (4.1)	16 (6.9)	
Wound, sternal dehiscence not related to infection	2 (0.5)	5 (2.1)	

*DCI*, Distressed Communities Index.
